# Development of a High-Throughput Screening Platform and a Pathogenesis Model for *Leishmania* Infection Based on Mouse Hepatic Organoids

**DOI:** 10.3390/ijms262412180

**Published:** 2025-12-18

**Authors:** María-Cristina González-Montero, Julia Andrés-Rodríguez, Miguel Criado, Sonia Andrés, Giulio Galli, Celia Fernández-Rubio, Yolanda Pérez-Pertejo, Rosa M. Reguera, Rafael Balaña-Fouce, Carlos García-Estrada

**Affiliations:** 1 Departamento de Ciencias Biomédicas, Facultad de Veterinaria, Universidad de León, Campus de Vegazana s/n, 24007 León, Spain; magom@unileon.es (M.-C.G.-M.); jandrr01@estudiantes.unileon.es (J.A.-R.); ggal@unileon.es (G.G.); cferrb@unileon.es (C.F.-R.); myperp@unileon.es (Y.P.-P.); rmregt@unileon.es (R.M.R.); 2 Instituto de Ganadería de Montaña, CSIC-Universidad de León, Finca Marzanas s/n, Grulleros, 24346 León, Spain; mcrib@unileon.es (M.C.); sonia.andres@eae.csic.es (S.A.); 3 Instituto de Biomedicina (IBIOMED), Universidad de León, Campus de Vegazana s/n, 24007 León, Spain

**Keywords:** organoids, liver, hepatic tissue, *Leishmania*, amastigote, drug discovery, high-throughput screening, pathogenesis model

## Abstract

The development of new alternative models is essential to overcome the limitations of traditional two-dimensional (2D) cell cultures and animal models. Three-dimensional (3D) models, such as organoids, better mimic the structural and functional complexity of mammalian organs, thereby reducing the ethical and economic issues related to animal experimentation. These systems provide more physiologically relevant environments, improving the accuracy of disease modeling and drug response prediction. In this context, we have developed mouse hepatic organoids from livers of adult BALB/c mice and characterized them by microscopy and transcriptional analysis. This model was applied to a robust and reproducible high-throughput screening (HTS) platform for testing cytotoxicity at the preclinical stage of drug discovery. In addition, mouse hepatic organoids were co-cultured with amastigotes of *Leishmania donovani* parasites to establish a model of host–parasite interaction, which was characterized by RNA-seq linked to differential expression analysis and cytokine production by the hepatic organoids. The findings provided in this work establish mouse hepatic organoids as an alternative model for drug discovery and pathogenesis studies.

## 1. Introduction

Biomedical research relies on a diverse array of tools and technologies to explore the mechanisms of health and disease at molecular, cellular, and organismal levels. In addition to molecular biology techniques (e.g., PCR, CRISPR/Cas9), advanced imaging systems (e.g., confocal and super-resolution microscopy), and high-throughput omics platforms (e.g., genomics, proteomics, metabolomics), three-dimensional (3D) culture models have emerged as pivotal tools by offering physiologically relevant models more faithful than traditional two-dimensional (2D) cultures, thus bridging the gap between traditional cell cultures and animal models [1]. Among 3D cultures, organoids, stem cell-derived mini-tissues that recapitulate much of the cellular diversity, architecture, and function of mammalian organs, are increasing their use in the study of development, genetic disorders, host–pathogen interactions, tumor biology and disease modeling under conditions that mimic in vivo physiology [2,3,4]. Furthermore, organoids are advancing precision medicine by serving as patient-specific platforms for drug screening, toxicity testing, and regenerative medicine applications [5]. However, despite these strengths, some limitations still remain, such as the lack of full cell-type complexity (immune, stromal, or vascular components) for some types of organoids, variability among batches, and issues related to maturation, scalability, and reproducibility [6,7,8].

Organoids have been developed from almost all tissues and organs from humans and different mammals [9,10]. Mouse hepatic/liver organoids are advanced 3D culture systems derived from primary hepatocytes or liver progenitor cells that retain many of the key functional liver attributes (e.g., production of albumin, expression of Arg1, gluconeogenic enzymes, cytochrome P450s) over extended culture periods, making them useful both for toxicity profiling and for modeling drug metabolism [11]. Mouse liver organoids have been used to test candidate drugs against nonalcoholic fatty liver disease [12] and along with their human counterparts, which have been used for drug validation and toxicity assessment [13], they can represent an alternative model for the screening of drugs against pathogens [5], mainly those zoonotic agents that still require the development of new drugs, as existing treatments often suffer from serious limitations, including the emergence of resistance and harmful side effects. Such is the case of *Leishmania*, a protozoan trypanosomatid parasite that requires a phlebotomine sandfly for transmission, and is responsible for leishmaniasis, one of the most prevalent zoonotic neglected tropical diseases with different clinical forms including cutaneous, mucocutaneous, and visceral leishmaniasis (VL) [14,15].

VL, also known as kala-azar, is the most severe manifestation with an estimated 50,000–90,000 new cases per year globally (mostly in Brazil, east Africa and India, though underreporting is significant), and is fatal if left untreated in over 95% of cases [16]. VL is caused by *Leishmania donovani* and *Leishmania infantum* (*Leishmania chagasi* in the New World), and is characterized by persistent irregular fever, hepatosplenomegaly, weight loss, pancytopenia, and hypergammaglobulinemia [14]. The parasites alternate between promastigotes (in sandfly vectors) and amastigotes (in mammalian macrophages), and pathology is largely driven both by parasite load and immune response [17]. Current treatments against VL rely on chemotherapy, with pentavalent antimonials (Sb^V^ complexes: sodium stibogluconate and meglumine antimoniate) still widely in use despite the severe side effects, the need for daily parenteral administration and the development of resistance [18,19,20]. Other drugs include amphotericin B (in its deoxycholate salt or liposomal formulations), paromomycin (in combinations with other drugs), pentamidine, and miltefosine, which are not exempt from side effects and other limitations. For example, amphotericin B can cause high fever, hypotension, nausea, headache, renal and hepatic failure, as well as allergic symptoms, and requires a cold chain to the point of application plus intravenous administration. Paromomycin must be administered intramuscular or intravenously and can cause nephrotoxicity, ototoxicity and hepatotoxicity. Pentamidine is rarely used due to its high toxicity, causing severe hypoglycemia, diabetes mellitus, nephrotoxicity, hypotension and myocarditis. Miltefosine, the first oral drug used for the treatment of leishmaniasis, despite its potent antileishmanial activity, is teratogenic and can produce severe gastrointestinal side effects [21,22].

Therefore, given the toxicity associated with existing antileishmanial therapies, along with the rise in drug-resistant *Leishmania* strains, there is an increasing demand for novel pharmacological agents to effectively treat VL. In this scenario, and given that liver is one of the target organs of *L. infantum* and *L. donovani*, we have developed mouse hepatic organoids to implement a robust and reproducible high-throughput screening (HTS) platform for testing cytotoxicity at the preclinical stage of drug discovery, together with a model of host–parasite interaction by means of co-culturing *L. donovani* amastigotes with mouse hepatic organoids. The findings provided in this work establish mouse hepatic organoids as an alternative model for drug discovery and pathogenesis studies.

## 2. Results

### 2.1. Development and Characterization of Mouse Hepatic Organoids

**Figure 1:**
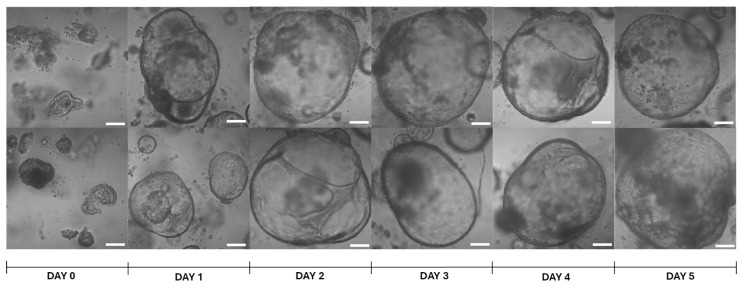
Representative images of brightfield microscopy of mouse hepatic organoids at different time-points. Day 0 refers to the time point of seeding the cell pellet, resuspended in Matrigel^®^ Matrix Basement Membrane Phenol-Red Free (Corning^®^, Discovery Labware Inc., Bedford, MA, USA), after cell passage. Pictures were taken with a Zeiss LSM 800 confocal microscope (Observer Z1, Carl Zeiss, Oberkochen, Germany) with 10× magnification. White bars denote 100 μm.

Mouse hepatic organoids were developed from BALB/c mouse adult livers as indicated in Section 4. After culture in HepatiCult™ Mouse Organoid Growth Medium (Stemcell Technologies™, Vancouver, BC, Canada), organoids were observed under the microscope for 5 days post-seeding, confirming their round shape along the culture time (Figure 1). If the organoids were not passaged, they showed signs of structural disruption starting on day 8–9.

**Figure 2:**
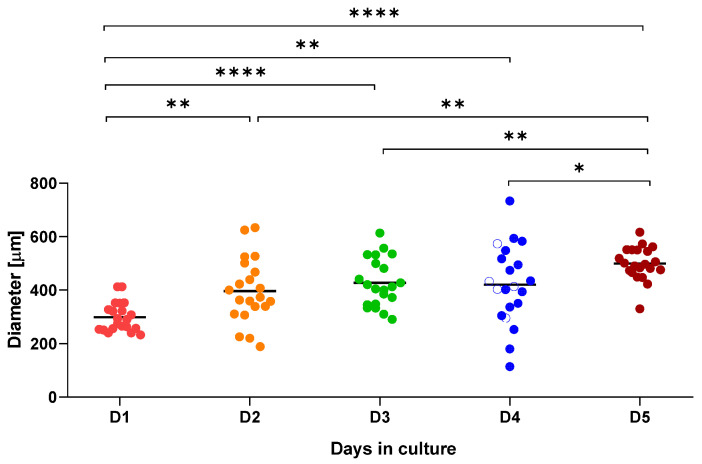
Graphical representation of mouse hepatic organoid growth over a 5-day (D1 to D5) period. Diameter (μm) was measured for 21 organoids at each time point. * *p* ≤ 0.05; ** *p* ≤ 0.01; **** *p* ≤ 0.0001.

The progress of organoid growth over time was assessed by measuring the largest diameter of 21 randomly selected organoids for 5 days, as described in Section 4. Mouse hepatic organoids exhibited rapid growth up to day 2 and displayed inter-replicate size variability until day 5, with significant differences in size relative to earlier time points (Figure 2).

After three passages, mouse hepatic organoids were stored in liquid nitrogen and revived 5 months later, showing a morphology similar to the parental organoids.

**Figure 3:**
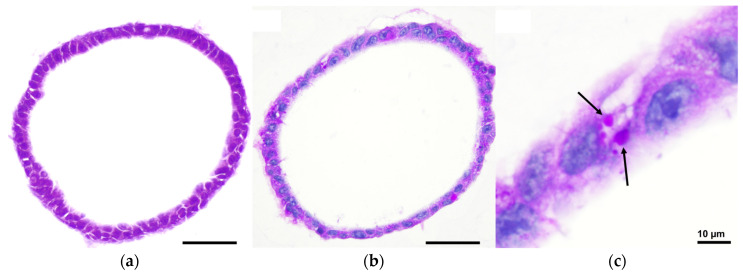
Brightfield microscopy images of mouse hepatic organoids after 7 days of in vitro culture: (**a**) H&E staining showing closed, spherical epithelial structures with a continuous monolayer of polygonal cells enclosing a central lumen; (**b**) Alcian Blue and PAS staining revealing cytoplasmic PAS-positive vacuoles indicative of glycogen accumulation; (**c**) Inset of panel b highlighting PAS-positive vacuoles (black arrows). Pictures were taken with a Nikon Eclipse Ni-E microscope (Nikon, Tokyo, Japan) equipped with a Prime BSI Scientific CMOS camera (Photometrics^®^ Prime BSI™, Scottsdale, AZ, USA). Scale bars = 50 µm, unless indicated otherwise.

To study the structural organization and cell composition of mouse hepatic organoids, organoid sections were first examined with hematoxylin and eosin (H&E) staining (Figure 3a). The organoids formed closed, spherical epithelial structures characterized by a continuous monolayer of polygonal cells enclosing a central lumen. These cells exhibited a cuboidal to columnar morphology, basophilic nuclei, and eosinophilic cytoplasm, consistent with hepatocyte-like characteristics. To further confirm its phenotypic resemblance with hepatocytes, Periodic Acid–Schiff (PAS) staining combined with Alcian blue was performed (Figure 3b,c). This revealed cytoplasmic PAS-positive vacuoles indicative of glycogen accumulation, supporting the metabolic resemblance of the organoid cells to hepatocytes.

**Figure 4:**
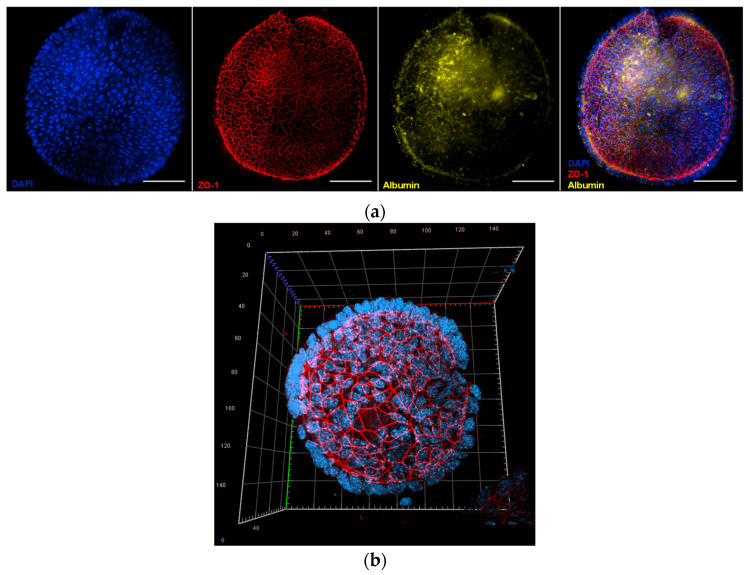
Fluorescence images of mouse hepatic organoids after 7 days of in vitro culture: (**a**) Representative image of mouse hepatic organoids stained with 4′,6-diamidino-2-phenylindole (DAPI, nuclei in blue color), and labelled with antibodies against Zonula Occludens 1 (ZO-1, tight junctions, red) and albumin (yellow). The merged image shows colocalization of these proteins. Images were taken with a Nikon Eclipse Ni-E microscope (Nikon, Tokyo, Japan) equipped with a Prime BSI Scientific CMOS camera (Photometrics^®^ Prime BSI™, Scottsdale, AZ, USA). Scale bars = 50 µm; (**b**) Three-dimensional confocal reconstruction of an organoid stained with DAPI and labelled with antibodies against ZO-1 (red), demonstrating apical-in polarity and basally localized nuclei. Confocal imaging was performed on a Zeiss LSM 800 (Observer Z1, Carl Zeiss, Oberkochen, Germany).

Immunofluorescence analysis (Figure 4a) was conducted to assess both tight junction integrity and hepatic function, using antibodies against Zonula Occludens 1 (ZO-1) and albumin. Narrow ZO-1-positive junctions were observed between adjacent cells, demonstrating well-established epithelial polarity, while albumin expression was detected in most cells at varying intensities, reflecting functional heterogeneity typical of hepatocytes [23]. Finally, confocal microscopy using a Zeiss LSM 800 confocal microscope (Observer Z1, Carl Zeiss, Oberkochen, Germany) was employed for three-dimensional reconstruction of the organoids (Figure 4b). ZO-1 localization at the luminal interface, together with basally positioned nuclei, confirmed apical-in polarity, further supporting the organoids structural and functional similarity to hepatocytes [24].

### 2.2. Comparative Transcriptional Analysis Between Mouse Hepatic Organoids and Mouse Hepatic Tissue

**Figure 5:**
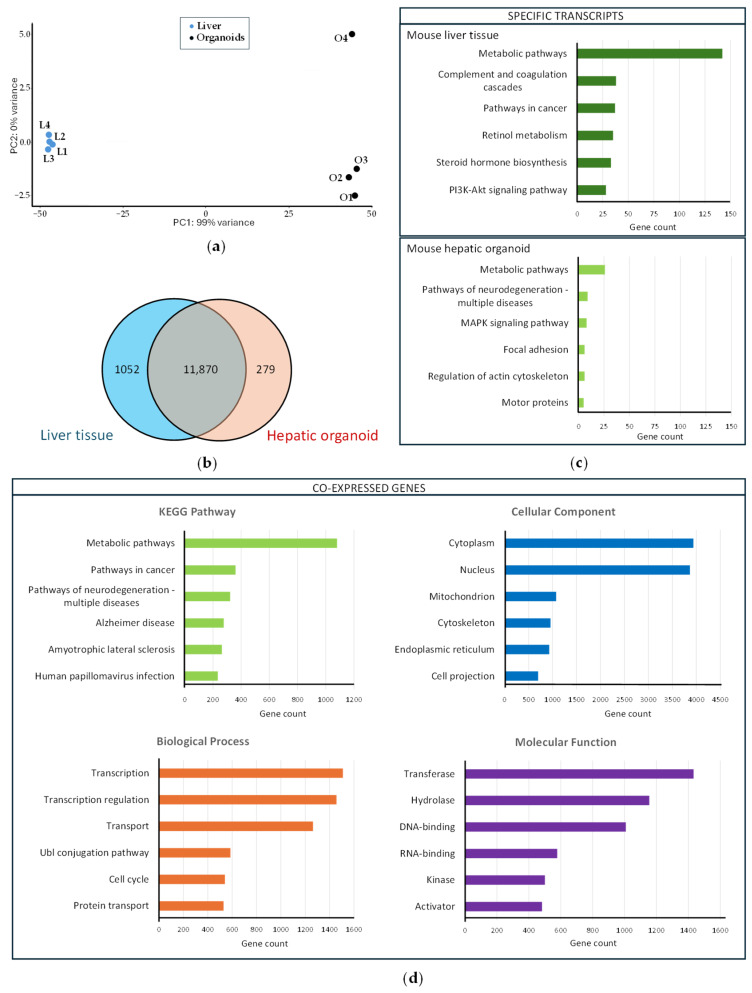
Transcriptional profiling of the liver and hepatic organoids: (**a**) Principal component analysis carried out with mouse liver samples (L1–L4, blue dots) and mouse hepatic organoids (O1–O4, black dots); (**b**) Venn diagram showing co-expressed genes among the mouse liver tissue and the mouse hepatic organoids (the blue circle includes the number of genes expressed in the liver tissue, whereas the red circle includes the number of genes expressed in hepatic organoids). The number of genes co-expressed both in the hepatic organoids and liver tissue is included in the intersection of the two circles; (**c**) Functional enrichment analysis of mouse liver tissue-specific transcripts (upper panel) and mouse hepatic organoid-specific transcripts (lower panel). The six most represented KEGG (Kyoto Encyclopedia of Genes and Genomes) pathways are represented as colored bars; (**d**) Functional enrichment analysis of the 11,870 genes co-expressed in the liver tissue and hepatic organoids. The six most represented pathways in each category (KEGG pathway, biological process, cellular compartment, and molecular function) are represented as colored bars.

RNA-seq analysis was carried out as described in Section 4 to compare gene expression profiles from mouse liver tissue and mouse hepatic organoids derived from this tissue. Principal component analysis (Figure 5a) resulted in statistically significant clusters (99% confidence intervals) for each sample type.

The transcriptional profile was analyzed in mouse hepatic organoids and liver tissue to confirm the similarity between organoids and liver. The analysis revealed that 11,870 genes were co-expressed in both hepatic organoids and liver, 1052 transcripts being liver tissue-specific and 279 transcripts being hepatic organoid-specific (Table S1) (Figure 5b). Functional enrichment analysis was performed with those specific genes using the DAVID free software (v2024q4) [25,26]. The most significant metabolic pathways (up to six), based on the KEGG results were identified for liver tissue- and hepatic organoid-specific transcripts (Figure 5c). Liver-specific transcripts were grouped in key biological pathways associated with mature liver functions, whereas organoid-specific transcripts were included in pathways associated with processes of structural organization, cytoskeleton remodeling, and signaling linked to proliferation and stress response.

Functional enrichment analysis using the DAVID free software (v2024q4) [25,26] was also carried out with the 11,870 co-expressed genes to identify the six most significant metabolic pathways, based on the KEGG results, as well as biological, cellular, and molecular categories (Figure 5d). Many of the genes co-expressed both in liver tissue and hepatic organoids are involved in metabolic pathways and are related to transcription and transcriptional regulation. Cytoplasm and nucleus are the most active cellular sites, and transferases and hydrolases are highly abundant.

**Figure 6:**
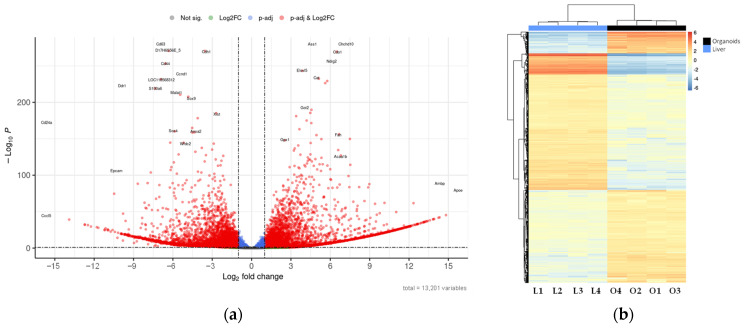
Differential expression between liver and hepatic organoids: (**a**) Volcano plot showing the genes with the highest differential expression between mouse hepatic organoids and mouse liver tissue. Plot represents statistical significance (*p*-value, Y axis) versus magnitude of change (fold change, X axis). Grey dots represent genes that show neither statistical significance nor differential expression. Blue dots indicate statistically significant genes without meaningful changes in expression. Green dots represent genes with expression changes that are not statistically significant. Red dots highlight genes that are both statistically significant and differentially expressed; (**b**) Heatmap showing the genes with the highest differential expression between mouse hepatic organoids (O1–O4) and mouse liver tissue (L1–L4). The applied filters are: Log2FC < −1 or >1, and q-value < 0.1. Color scale indicates gene expression levels from low (blue) to high (red). Dendrograms reflect hierarchical clustering based on sample similarity.

DESeq2 analysis of differential expression identified 4183 upregulated (Table S2) and 3494 downregulated (Table S3) genes in liver versus hepatic organoids, which can be visualized in a Volcano plot (Figure 6a) and a heatmap (Figure 6b), showing distinct expression patterns based on significant fold changes and q-values.

The six most upregulated and downregulated genes in liver versus hepatic organoids are included in Table 1 and Table 2, respectively.

The higher expression of genes related to key metabolic functions, cell regulation and detoxification in the mature liver in in vivo tissue (Table 1) suggests that the organoids lack the maturity or the microenvironmental cues necessary to fully perform these functions. On the other hand, downregulation in the liver tissue (upregulation in organoids) of genes associated with processes opposite to mature differentiation, such as cell adhesion, morphogenesis, and proliferation (Table 2), suggests a less mature state of the organoids.

### 2.3. Development of an HTS for Drug Discovery Purposes Based on Mouse Hepatic Organoids

In order to implement a robust 3D platform for in vitro safety assessment during the screening of compound libraries in the drug discovery process, mouse hepatic organoids were applied to a 384-well HTS platform. The organoid morphology and growth in 384-well plates was similar to that observed in 24-well plates and therefore, cytotoxicity tests were conducted in this platform. With this purpose, organoids were cultured in 384 well-plates for four days and incubated for 72 h with either 0.03% H_2_O_2_ (cytotoxicity control) or 0.2% DMSO (viability control). Cell viability was assessed by means of the fluorescence emitted by organoids after 24 h in the presence of alamarBlue™ HS staining reagent (Invitrogen™, Thermo Fisher Scientific™, Waltham, MA, USA). Assay robustness and reliability were assessed by calculating key statistical metrics as indicated in Section 4. On average, a remarkable Z′-factor of 0.7 was obtained with four replicates (Table 3), thus confirming mouse hepatic organoids as a 3D robust HTS platform for drug discovery applications.

**Figure 7:**
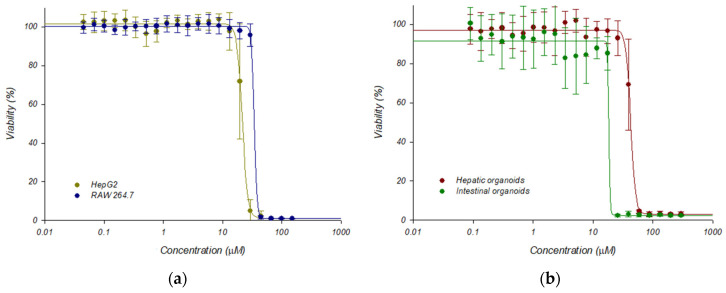
Dose response curves adjusted with the Sigma Plot 10.0 statistical package showing the cytotoxicity of 5-nitro-2-furonitrile: (**a**) Cytotoxicity on established HepG2 and RAW-264.7 cell lines; (**b**) Cytotoxicity on mouse hepatic and intestinal organoids. Dose–response curves were performed plotting the percentage of viability obtained after 72 h of incubation in the presence of 5-nitro-2-furonitrile. Data represent the mean ± standard deviation of three different experiments carried out in triplicate.

This platform was validated with 5-nitro-2-furonitrile, a nitrofuran compound that was previously characterized by our group in terms of antileishmanial and cytotoxic activities [27]. As a result, dose–response curves were obtained, testing different concentrations of the above-mentioned molecule in the HTS platform containing mouse hepatic organoids, and were compared to data provided by classical 2D cultures of HepG2 and RAW 264.7 cells, and with another 3D culture of mouse intestinal organoids, the latter recently developed by our group [28] (Figure 7). Cytotoxicity was determined using the alamarBlue™ cell viability reagent (Invitrogen, Fisher Scientific International Inc., Waltham, MA, USA) as indicated in Section 4, and the CC_50_ values, calculated with the SigmaPlot 10.0 software package, were 19.73 ± 0.40, 34.44 ± 0.91, 44.88 ± 0.99, and 22.53 ± 1.17, for HepG2 cells, RAW 264.7 cells, hepatic organoids and intestinal organoids, respectively. The CC_50_ values between HepG2 cells and mouse hepatic organoids were found to be significantly different (*p* < 0.05).

### 2.4. Co-Culture of Mouse Hepatic Organoids and L. donovani-iRFP Amastigotes as a Model of Host–Parasite Interaction and Transcriptome-Wide Analysis of the Effect of L. donovani-iRFP on Mouse Hepatic Organoids

**Figure 8:**
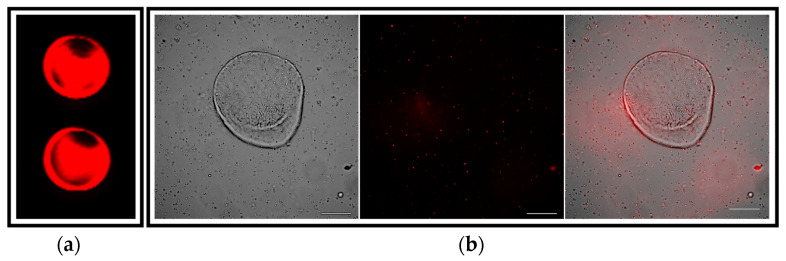
Visualization of the co-culture between mouse hepatic organoids and *L. donovani*-iRFP amastigotes: (**a**) Near-infrared fluorescence emitted by viable *L. donovani*-iRFP after 24 h of growth. Two representative wells (96-well plate) are shown. Fluorescence was measured at 700 nm with an Odyssey infrared imaging system (Li-Cor, Lincoln, NE, USA); (**b**) Microscopy images showing the co-culture of one representative mouse hepatic organoid and the *L. donovani*-iRFP amastigotes (left panel), the infrared emitted by amastigotes visualized with the 740 nm channel (middle panel) and the merged image (right panel). Pictures were taken with a Nikon Eclipse Ni-E microscope (Nikon, Tokyo, Japan) equipped with a Prime BSI Scientific CMOS camera (Photometrics^®^ Prime BSI™, Scottsdale, AZ, USA). White bars denote 100 μm.

To establish an in vitro pathogenic model of the *Leishmania* infection, mouse hepatic organoids were co-cultured with axenic *L. donovani*-iRFP amastigotes for 24 h. Viability of the parasites was confirmed by measuring the near-infrared fluorescence emitted by the infrared fluorescent protein (iRFP) present in the amastigotes just before their addition to the organoids (Figure 8a). The co-culture was visualized under the microscope (Figure 8b), thus confirming the interaction of viable parasites with the mouse hepatic organoids.

**Figure 9:**
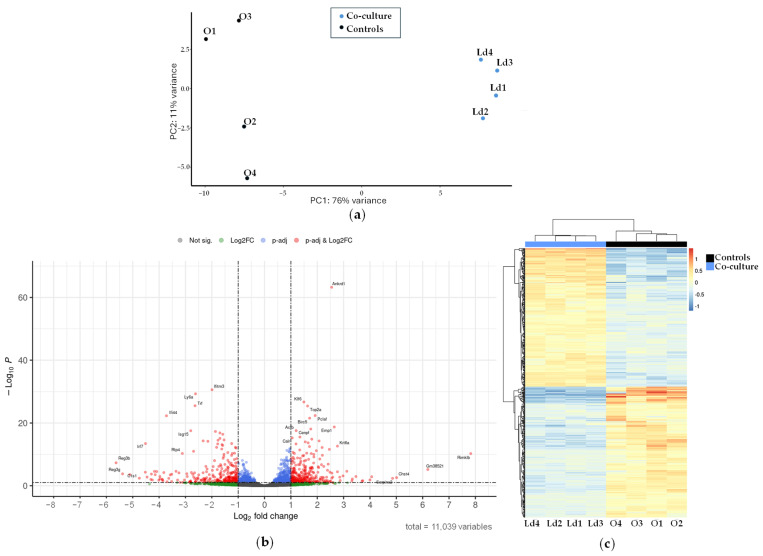
Transcriptional analysis of the mouse hepatic organoids co-cultured with *L. donovani*-iRFP: (**a**) Principal component analysis carried out with samples of mouse hepatic organoids (O1–O4, black dots) and mouse hepatic organoids co-cultured with *L. donovani*-iRFP amastigotes (Ld1–Ld4, blue dots); (**b**) Volcano plot showing the genes with the highest differential expression between mouse hepatic organoids and mouse hepatic organoids co-cultured with *L. donovani*-iRFP amastigotes. Plot represents statistical significance (*p*-value, Y axis) versus magnitude of change (fold change, X axis). Grey dots represent genes that show neither statistical significance nor differential expression. Blue dots indicate statistically significant genes without meaningful changes in expression. Green dots represent genes with expression changes that are not statistically significant. Red dots highlight genes that are both statistically significant and differentially expressed; (**c**) Heatmap showing the genes with the highest differential expression between mouse hepatic organoids (O1–O4) and mouse hepatic organoids co-cultured with *L. donovani*-iRFP amastigotes (Ld1–Ld4). The applied filters are: Log2FC < −1 or >1, and q-value < 0.1. Color scale indicates gene expression levels from low (blue) to high (red). Dendrograms reflect hierarchical clustering based on sample similarity.

Differences in gene expression profiles between mouse hepatic organoids and mouse hepatic organoids co-cultured with *L. donovani*-iRFP amastigotes were determined by RNA-seq as described in Section 4. Principal component analysis (Figure 9a) resulted in statistically significant clusters (76% confidence intervals) for each sample type, with no outliers identified.

From the 11,039 genes co-expressed in both conditions (Table S4), differential expression analysis using DESeq2 identified 295 upregulated (Table S5) and 280 downregulated (Table S6) genes when *L. donovani*-iRFP was in co-culture with organoids. These results are visualized in a Volcano plot (Figure 9b) and a heatmap (Figure 9c), showing distinct expression patterns based on significant fold changes and q-values.

The six most upregulated and downregulated genes when *L. donovani*-iRFP was co-cultured with mouse hepatic organoids are included in Table 4 and Table 5, respectively.

The simultaneous overexpression of Klf6, Birc5, Top2a, Pclaf, Ankrd1, and Emp1 (Table 4) suggest that the organoid, in response to the parasite, is undergoing stress, damage, or tissue remodeling, with the hepatic 3D structure attempting to resist or contain the infection through cell proliferation, inhibition of apoptosis, and mechanisms of regeneration or fibrosis. On the other hand, downregulation of genes such as Ifi44, Isg15, Ly6a, Trf, Ifitm3, and Gsta3 (Table 5) suggests a suppression of pathways related to innate immunity, interferon signaling, oxidative stress response, and iron metabolism inducing an immunosuppressive and pro-parasitic environment in the liver, likely aiding its persistence and replication.

**Figure 10:**
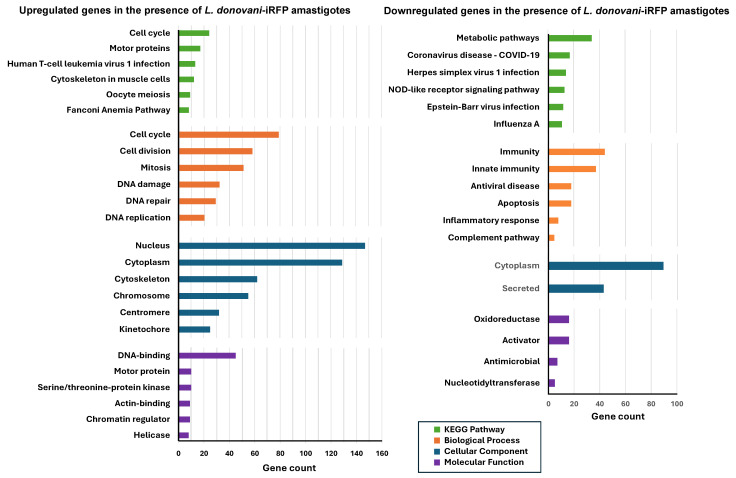
Functional enrichment analysis of the differentially expressed genes in mouse hepatic organoids co-cultured with *L. donovani*-iRFP. The left panel shows the analysis of the 295 genes of mouse hepatic organoids that are upregulated in the presence of *L. donovani*-iRFP amastigotes, whereas the right panel shows the analysis of the 280 genes of mouse hepatic organoids that are downregulated in the presence of *L. donovani*-iRFP amastigotes. The six most represented pathways in each category (KEGG pathway, biological process, cellular compartment, and molecular function) are represented as colored bars (only two chart records and four chart records were provided for the cellular component and molecular function categories, respectively, by the DAVID free software (v2024q4) during the analysis of downregulated genes).

For a detailed characterization of the changes in the gene expression profile of the organoids due to the presence of *L. donovani*-iRFP amastigotes, functional enrichment analysis of the 295 upregulated and 280 downregulated genes included in Tables S5 and S6 was performed using the DAVID free software (v2024q4) [25,26]. The most significant metabolic pathways (up to six), based on the KEGG results, as well as biological, cellular, and molecular categories were identified for the upregulated and downregulated genes (Figure 10).

In the presence of *L. donovani*-iRFP amastigotes, organoids exhibited high expression of genes associated with cell cycle and division, proliferation and DNA repair. The nucleus was identified as the most active cellular site, while genes encoding DNA-binding proteins were abundantly transcribed. On the other hand, *L. donovani*-iRFP amastigotes repressed genes related to the immune response, complement pathway and apoptosis. The cytoplasm was identified as the main cellular compartment where the majority of gene products were downregulated, while oxidoreductases and activators were repressed (Figure 10). These results confirm the pattern shown by the six most upregulated and downregulated genes included in Table 4 and Table 5.

### 2.5. L. donovani-iRFP Impairs the Ability of Mouse Hepatic Organoids to Participate in the Immune Response and Leads to a Reduction in the Production of Nitric Oxide (NO) and Pro-Inflammatory Cytokines

To further characterize the effect of *L. donovani*-iRFP on the host’s immune response, a detailed analysis of the differential expression of genes involved in this process was conducted, focusing on the 295 upregulated and 280 downregulated genes listed in Tables S5 and S6. *L. donovani*-iRFP upregulated 21 genes (Table S7) and downregulated 71 genes (Table S8) encoding cytokines and other proteins involved in the inflammatory process and immune response. The analysis of the overexpressed genes suggests the activation of the innate and adaptive immune responses involving NK and T cells (e.g., Il15, Il33, Klrc1, Lgals1, Fos, Egr1), acute inflammatory signaling (via MAPK, AP-1) (e.g., Dusp1, Dusp10, Birc5, Inhba, Psip1), attempts at tissue repair and modulation of inflammation (e.g., Tnc, Fst, Serpine2, Edn1), as well as possible parasite evasion mechanisms through the induction of immunoregulatory genes (e.g., Lgals1, Serpine2, Inhba). On the other hand, the analysis of the downregulated genes indicates a suppression of the antiviral and type I interferon response, with a massive repression of the IFN-I axis that is a key defense pathway against many intracellular pathogens (e.g., Stat1, Stat2, Irf7, Irf9, Isg15, Ifi35, Oas1a/b/2, Rsad2, Ddx60, Dhx58, Ifit1, Ifitm3, Clec2d), altered adaptive immunity and antigen processing and presentation (e.g., B2m, Psmb8, Psme1, H2-K1), suppression of apoptosis, autophagy cellular damage and stress allowing more permissive environments that favor parasite replication and survival (e.g., Casp4, Casp12, Bmf, Ddit4, Ern1, Gadd45g, Chac1), and suppression of the Complement system impairing direct elimination of the parasite (e.g., C3, C4b, C4bp, Cfb, Cfh, C1ra, C1s1).

**Figure 11:**
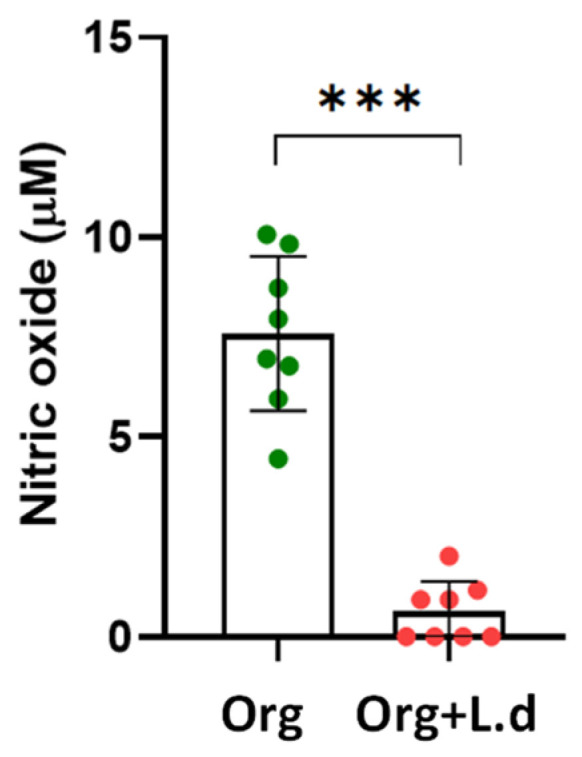
NO production (μM) by mouse hepatic organoids (Org, green dots) after 24 h of co-culture with *L. donovani*-iRFP amastigotes (Org + L.d, red dots). Results show the mean ± SD of eight values obtained from four independent experiments carried out in duplicate. *** *p* ≤ 0.001.

To confirm the effect of *L. donovani* in the immunological response exerted by the mouse hepatic organoid, NO production was assessed after 24 h of co-culture by the Griess reaction. Strikingly, the presence of *L. donovani*-iRFP amastigotes gave rise to a dramatic decrease in NO levels produced by the organoids (Figure 11), which was not related to a significant modification in the expression of the Nos2 gene encoding the inducible nitric oxide synthase (iNOS), as inferred by the data included in Supplementary Tables S4–S6.

**Figure 12:**
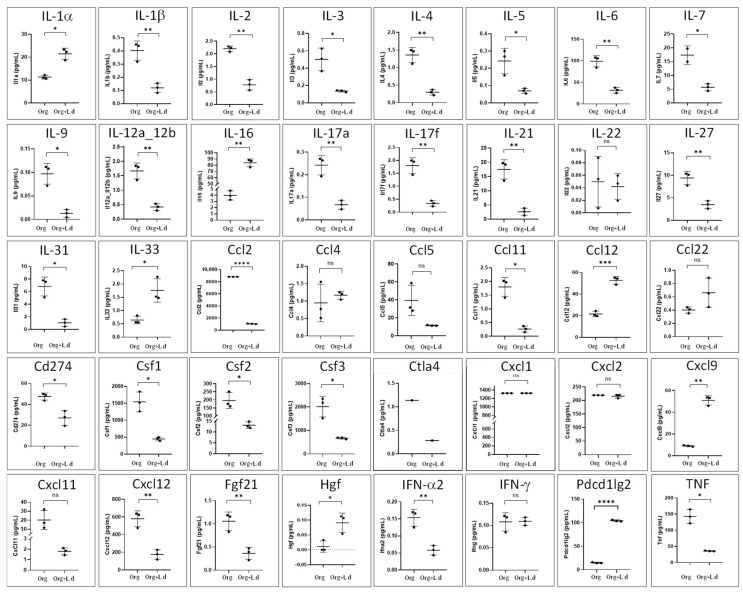
Production of cytokines (pg/mL) by mouse hepatic organoids (Org) after 24 h of co-culture with *L. donovani*-iRFP amastigotes (Org + L.d). Results show the mean ± SD of three technical replicates. ns, non-significant; * *p* ≤ 0.05; ** *p* ≤ 0.01; *** *p* ≤ 0.001; **** *p* ≤ 0.0001.

Cytokine levels were also determined in the culture supernatants of organoids grown in the absence and presence of *L. donovani*-iRFP amastigotes (24 h co-culture). With this purpose the Olink^®^ Target 48 Mouse Cytokine Panel (Olink Proteomics, Uppsala, Sweden) was used. Results were provided for 40 cytokines (Figure 12), which pointed out to a globally suppressive and reparative immunological pattern characterized by a strong attenuation of both Th1- and Th17-associated inflammatory pathways (exemplified by a reduction in the levels of IL-1β, IL-6, TNF, IL-17A, IL-17f, IL-21, and IL-12), a repression of classic Th2-type activation (exemplified by a decrease in IL-5, IL-9 and IL-21 levels, although the increase in IL-33, an alarmin associated with tissue repair and a Th2-type response, suggests a shift toward a repair/tolerance state), a systemic immunosuppressive effect associated with the decrease in factors that maintain the proliferation and survival of T and B lymphocytes (exemplified by IL-2, IL-3 and IL-7), a decrease in the production of factors that promote the proliferation of macrophages, granulocytes, and monocytes (exemplified by Csf1, Csf2 and Csf3), a selective modulation of leukocyte traffic through the reduction in the overall recruitment of inflammatory cells (monocytes, eosinophils, Th1/Th17 lymphocytes) (exemplified by the reduction in Cxcl12, Cxcl11, Ccl2 and Ccl11), a selective tuning of inhibitory signaling pathways that may favor immune tolerance rather than complete immune suppression (decrease in Ctla4, Cd274 and increase in Pdcd1lg2), and increased levels of molecules associated with tissue repair, homeostasis, and tolerance (exemplified by HGF, IL-16 and IL-33).

These results confirm that mouse hepatic organoids respond to *L. donovani* infection through a profound reprogramming of its gene expression profile, characterized by protection from the invasion and coordinated downregulation of genes involved in innate and adaptive immune responses, inflammation, complement activation and apoptosis.

## 3. Discussion

Organoids represent a very important tool for biomedical research, since they allow for the modeling of human and animal organs for drug discovery and to study diseases with greater precision, thereby reducing the use of animals and improving the prediction of treatment responses [29,30]. In this scenario, in order to obtain an HTS platform useful for drug discovery purposes and a model for the characterization of host–pathogen interactions, we have developed and characterized mouse hepatic organoids from adult mouse livers. The spherical morphology, progressive growth during the first days of culture, and preservation of its integrity after long-term storage are consistent with previous studies on liver organoids obtained from mammalian progenitor cells or hepatocytes [31,32,33]. The presence of a continuous epithelial layer, the apical-in polarity confirmed by ZO-1, and the expression of functional markers such as albumin reinforce the notion that this system reproduces essential characteristics of mature hepatocytes, as observed in liver organoids from other species [23,24].

The organoids developed in this work have been grown in HepatiCult™ Mouse Organoid Growth Medium (HepatiCult™ mouse OGM, Stemcell Technologies™, Vancouver, BC, Canada). According to the manufacturer, organoids grown in this medium feature an epithelium expressing genes marking hepatic stem and progenitor cells (PROM1, AXIN2, SOX9 and CD44), ducts (KRT19 and HNF1b) and hepatocytes (HNF4a, AFP). Expression of these genes and others selectively expressed in the intrahepatic bile duct, such as *cftr* [34] (Table S1), as well as the glycogen accumulation observed by PAS, indicate that the 3D system developed in this work contains a heterogeneous population similar to the hepatic microenvironment in vivo, which is consistent with what has been described in organotypic cultures derived from adult tissue [35,36].

Analysis of the transcriptional profile suggested that hepatic organoids do not fully recapitulate certain metabolic and signaling pathways characteristic of the mature liver, and indicated that organoids maintain transcriptional programs compatible with a more immature or dynamic cellular state compared to native liver tissue. These observations are consistent with previous studies showing that hepatic organoids exhibit incomplete maturation of metabolic networks, and display transcriptional signatures resembling fetal or neonatal hepatocytes [37]. Functional enrichment analyses showed that genes co-expressed in the hepatic and organoids are primarily clustered in metabolic pathways and transcriptional regulatory processes, consistent with the liver’s role as a central organ of metabolism. They also highlight the cytoplasm and nucleus as dominant locations, as well as the abundance of transferases and hydrolases, key enzymes in drug metabolism and detoxification. These findings align with previous studies describing hepatic co-expression networks heavily enriched in lipid metabolism and transcriptional regulation [38]. The abundance of transferases and hydrolases is consistent with the role of these enzymes in xenobiotic metabolism and maintenance of hepatic homeostasis [39].

From a technological point of view, adapting mouse hepatic organoids to a miniaturized 384-well format demonstrated their suitability for HTS platforms, and therefore, for their application for drug discovery purposes. The high Z′-factor obtained (0.7) falls within the range considered optimal for robust pharmacology assays as reported for other HTS platforms [40,41], and suggests that this model can be reliably used in toxicity studies and early selection of candidate compounds. Our group recently implemented this approach using sheep and mouse duodenum organoids [28,42], thus proving the suitability of 3D organoid platforms. As reported for sheep duodenum organoids, data from the Z′ factor test provided by mouse hepatic organoids, although still robust, suggested a higher variability than classical 2D models. The Z′-factor is a statistical parameter commonly used to evaluate the quality and reliability of high-throughput screening assays, with values above 0.5 considered indicative of a robust and reproducible platform. The slightly higher variability observed in 3D organoids likely reflects the more complex cellular architecture and heterogeneous microenvironments present in these systems, which better mimic physiological conditions. Consequently, while 3D organoids may show greater inter-well variability than 2D monolayers, they provide a more physiologically relevant response, capturing drug metabolism, cellular interactions, and tissue-specific responses that are often absent in conventional 2D cell lines. Validation using 5-nitro-2-furonitrile confirmed this pattern: although mouse hepatic organoids were less sensitive than 2D models (HepG2 and RAW 264.7 cells), this aligns with the growing evidence that organoids more faithfully recapitulate in vivo pharmacological behavior [43,44]. In addition, mouse hepatic organoids were less sensitive to 5-nitro-2-furonitrile than mouse intestinal organoids, probably due to metabolic differences between these two types of organoids. In fact, several studies have shown that hepatic and intestinal organoids have markedly different metabolic profiles after exposure to different compounds [45,46].

One of the most relevant results of this study is the characterization of a 3D organoid model for the initial analysis of host–parasite interactions during *L. donovani* infection. To the best of our knowledge, this is the first report of organoids being used in combination with *Leishmania* to characterize the interaction between host and pathogen. The closest approach so far in the literature described the exposure of 3D hepatic spheroids of canine hepatocytes to *L. infantum* promastigotes, showing that hepatocytes responded to the parasite by increasing NO production and modulating TLR receptors and cytokines [47]. The limitation of using single-cell-type 3D structures co-cultured with promastigotes was overcome in our model, in which the integration of amastigotes within the extracellular matrix without compromising parasite viability, confirmed the system’s suitability for studying invasion, persistence, and immunomodulation in a multi-cell-type hepatic 3D environment. This is consistent with previous works demonstrating the usefulness of organoids in modeling complex infections, including those caused by intracellular parasites [48,49,50,51].

Transcriptional analysis of the mouse hepatic organoids after co-culturing with *L. donovani*-iRFP amastigotes revealed a profound reprogramming of the hepatic epithelium in response to the parasite, characterized simultaneously by activation of cell proliferation and tissue repair pathways, along with modulation of inflammatory immune response, a pattern in agreement with known evasion strategies of *Leishmania* [52]. The pattern of upregulated genes due to the presence of the parasite suggests activation of regeneration, cell survival, and tissue remodeling and repair programs, consistent with early hepatocyte responses to damage [53,54,55,56]. In addition, overexpression of genes such as Il33 and Il15 may play a particularly relevant role as modulators of inflammation. It has been reported that IL-33 could repress the Th1 response of the hepatic immunological reaction against *L. donovani* [57], and that *L. donovani* induces the production of this cytokine in macrophages via the cAMP/EPAC/calcineurin pathway, which favors an immunosuppressive environment and parasite survival [58]. The overexpression of Il15 could reflect an autocrine or paracrine signaling mechanism by hepatocytes to promote cell survival and modulate local immunity, enhancing their resistance to parasite-induced damage while maintaining resident lymphocyte homeostasis. Studies have shown that hepatocytes produce IL-15 as an essential signal for the maintenance of NK and NKT cells in the liver [59]. Furthermore, the trans-presentation of IL-15 through its α receptor in liver cells has antifibrotic effects, suggesting that IL-15 may also be involved in tissue repair or remodeling programs [60]. In parallel, the marked repression of interferon-dependent genes reflects an inhibition of the IFN-I axis that favors the intracellular survival of the parasite by limiting the activation of antimicrobial pathways. Previous studies have suggested that *Leishmania* could exploit the type I IFN signaling pathway to negatively modulate the early host immune response, thereby favoring its survival [61,62]. Furthermore, the decrease in the expression of genes involved in antigen processing and presentation and those associated with apoptosis, autophagy, cellular stress, or damage suggests a broader disruption of cellular defenses, reducing parasite clearance and creating a more favorable cellular environment for its replication. This type of global modulation of the cellular immune system has been described as part of the immune subversion mechanisms used by *Leishmania* to avoid activating inflammatory responses and facilitate intracellular survival [63]. On the other hand, it is well-known that *Leishmania* can recruit host complement regulators to inactivate key components of this system and avoid complement-mediated lysis [64,65]. The downregulation of complement components observed in mouse hepatic organoids during *L. donovani*-iRFP co-culture suggests that the parasite not only evades the complement system at the protein level, but can also suppress the synthesis of complement components and regulators in hepatocytes, reducing the effectiveness of the innate response and creating a more favorable environment for its survival. Thus, genetic repression of complement components in the host could enhance this evasion effect, reducing the elimination of the parasite by direct lysis. In summary, transcriptional analysis revealed a profound reprogramming of the immunological response, cellular signaling, and the stress/inflammatory pathways, likely reflecting immune evasion strategies employed by the parasite. Therefore, *Leishmania* triggers a localized inflammatory response in hepatic organoids, but blocks key mechanisms of the antiviral, adaptive, innate and apoptotic immune systems, thereby promoting a cellular environment that allows its persistence and replication.

Data from transcriptomics analyses illustrate that the transcriptional reprogramming observed during *L. donovani* infection represents a fundamentally different biological stress compared to the transcriptional differences between native liver and hepatic organoids. While the liver–hepatic organoid contrast is dominated by metabolic and differentiation-related changes reflecting adaptation to in vitro culture, infection induces a pathogen-driven response characterized by stress adaptation, survival, cell cycle regulation, and selective modulation of interferon-stimulated and innate immune pathways. These findings emphasize that, although both types of comparative conditions involve cellular stress, the nature and consequences of the transcriptional programs are distinct.

Consistent with the transcriptomic results of the co-culture with *L. donovani*-iRFP, the significant reduction in NO production is a key finding. NO suppression represents one of the best-documented immune evasion mechanisms in *Leishmania*, essential for avoiding macrophage-mediated destruction [66]. In our model, NO is produced directly by hepatocytes and not by macrophages. In the liver, during proinflammatory reactions, arginase and iNOS compete for L-arginine [67]. Although iNOS expression remains stable in mouse hepatic organoids infected with *L. donovani*-iRFP, the strong reduction in NO production suggests a post-transcriptional blockade of the enzyme’s activity. One plausible mechanism for this blockade is competition for substrate: arginase (of parasitic or host origin) could consume the available L-arginine, reducing its availability for iNOS and thus limiting the generation of effective NO, as it has been previously described [68].

The cytokine profile of the mouse hepatic organoids after the co-culture with *L. donovani*-iRFP confirmed a generally anti-inflammatory and reparative environment, with a marked decrease in Th1 and Th17 axes, as well as a reduction in cytokines that regulate lymphocyte and myeloid cell proliferation and activity, which is coincident with the signaling mechanisms described for *Leishmania* [69]. This modulation also resembles the immunoregulatory patterns observed in hepatic models of VL, where the infection is associated with IL-10 and IL-27-mediated suppression of Th1 and Th17 responses, reduced macrophage activation, and decreased production of effector cytokines, which facilitates parasite persistence [70]. These results indicate that *L. donovani* induces a controlled, anti-inflammatory, and reparative immunological environment in hepatic organoids, which likely contributes to the parasite’s persistence by reducing excessive inflammation and NO production, and preserving cell viability and tissue integrity. Although there is no solid evidence that *Leishmania* infects hepatocytes, several signals and factors must be produced by the parasite inducing the profound transcriptional and functional changes observed in these cells. This suggests that hepatocytes respond to signals derived from the parasite or to systemic immune mediators, rather than to a direct infection, as has been previously reported [71].

## 4. Materials and Methods

### 4.1. Preparation and Culture of Mouse Hepatic Organoids

Mouse hepatic organoids were cultured following protocols based and adapted from the HepatiCult™ Organoid Growth Medium (Mouse) and Mouse Hepatic Progenitor Organoid Culture [72,73]. To obtain hepatic progenitor cells, BALB/c mice were used. Briefly, after humane euthanasia, liver was removed and rinsed twice in ice-cold DMEM/F-12 (Gibco, Life Technologies Limited, Paisley, UK) supplemented with 15 mM HEPES. Tissue was cut into segments and incubated with Tissue Dissociation Cocktail for six 20-min digestion cycles. The supernatant was sequentially filtered through 70 µm and 37 µm (reversible) strainers, and the final flow-through was discarded. The reversible 37-µm strainer was then inverted and rinsed with Advanced DMEM/F-12 (Gibco, Life Technologies Corporation, Grand Island, NE, USA) to recover the retained fraction. This eluate was centrifuged at 290× *g* for 5 min, and the pellet was resuspended in Matrigel^®^ matrix (Corning^®^, Discovery Labware Inc., Bedford, MA, USA). The cell suspension (35 µL) was dispensed onto pre-warmed 24-well plates, where they were allowed to solidify as domes. The plates were then incubated at 37 °C for 10 min to promote matrix polymerization, and 500 µL of HepatiCult™ Mouse Organoid Growth Medium (HepatiCult™ mouse OGM, StemCell Technologies™, Vancouver, BC, Canada) were carefully overlaid. Cultures were maintained at 37 °C with 5% CO_2_, and medium was replaced every 2–3 days. All animal experiments complied with Spanish and European Union regulations (RD 53/2013 and 2010/63/EU) and were approved by the institutional Ethics Committee (Project license OEBA-ULE 010-2023).

Organoids were subcultured once a week. After removing the culture medium, the domes were incubated for 1 min with 800 µL of ice-cold advanced DMEM/F-12, and the matrix was disrupted by gentle pipetting. The resulting suspension was collected into a 15-mL conical tube, and the wells were rinsed with an additional 500 µL of medium to maximize recovery. Samples were centrifuged at 290× *g* for 5 min and the pellet obtained was resuspended and plated following the procedure described above.

To preserve the organoids for long-term storage, two wells containing approximately 200 organoids each, were collected. The matrix was dissociated using 1 mL of CryoStor^®^ CS10 (StemCell Technologies™, Vancouver, BC, Canada), and the organoids in the cryopreservation medium were introduced in cryovials, which were stored in liquid nitrogen for future use.

The 384-well assay was adapted from the protocol described in [42] with slight modifications. Following medium removal, organoids were recovered from the extracellular matrix for passaging, and the resulting pellet was resuspended in 3.2 mL of a 1:1 mixture of Matrigel^®^ matrix (Corning^®^, Discovery Labware Inc., Bedford, MA, USA) and HepatiCult™ Mouse OGM (StemCell Technologies™, Vancouver, BC, Canada). Using a precooled multichannel dispenser, 8 µL of this suspension was seeded into pre-chilled 384-well plates. The plates were gently swirled by hand to evenly spread the matrix and incubated at 37 °C for 10 min to allow polymerization. Subsequently, 32 µL of HepatiCult™ Mouse OGM (StemCell Technologies™, Vancouver, BC, Canada) was added to each well, while 100 µL of sterile water was placed in the outer wells to keep the plate moist.

### 4.2. Histochemical and Immunofluorescence Staining

For histochemistry and immunofluorescence assays, seven-day-old organoids were fixed using methanol-free paraformaldehyde (Thermo Fisher Scientific, Waltham, MA, USA) at 4% in phosphate-buffered saline (PBS) for 30 min at room temperature (RT). Organoids were then removed from Matrigel^®^ domes using ice-cold PBS, and washed twice in PBS. Organoids were handled using wide-bore (trimmed) pipette tips, to reduce shear stress.

For sectioning and histochemical staining, organoids were embedded in agarose following a previously described procedure [74]. Briefly, organoids were resuspended in 2% high-purity agarose (Biotools, Madrid, Spain) dissolved in PBS and allowed to cool to 60 °C in a 50 mL Falcon tube. After cooling on ice, agarose-embedded organoids were carefully removed from the tube and further fixed in 70% ethanol, then processed for conventional paraffin embedding. Sections of 3 µm thickness were cut and mounted onto poly-L-lysine–coated slides (SuperFrost Plus Adhesion slides, Thermo Fisher Scientific, Waltham, MA, USA). Sections were stained with H&E or Alcian Blue (pH 2.5) followed by PAS to detect glycogen, as well as acidic and neutral glycoconjugates, including mucins and glycoproteins.

For immunofluorescence labeling of organoids, permeabilization and blocking were performed by incubating organoids in 0.1% Triton™ X-100 (Sigma-Aldrich, St. Louis, MO, USA) and 3% bovine serum albumin (BSA; Roche Diagnostics, Mannheim, Germany) in PBS for 1 h at RT on a plate shaker at low speed to maintain organoid suspension. Subsequently, organoids were incubated with rat anti-ZO-1 Alexa Fluor 594-conjugated antibody (R40.76, Santa Cruz Biotechnology, Dallas, TX, USA) and rabbit monoclonal anti-albumin Alexa Fluor 750-conjugated antibody (Novus Biologicals, Centennial, CO, USA), both at a 1:50 dilution in PBS containing 3% BSA and 0.1% Triton™ X-100 for 2 h at RT under gentle agitation. Following primary antibody incubation, organoids were washed five times with PBS containing 0.05% Tween 20 and resuspended in Fluoroshield™ mounting medium with DAPI (4′,6-diamidino-2-phenylindole; Sigma-Aldrich, St. Louis, MO, USA). Organoids were then whole-mounted onto poly-L-lysine-coated slides (Sigma-Aldrich, St. Louis, MO, USA), using #1 coverslips as spacers to avoid compression.

Imaging was performed using a Nikon Eclipse Ni-E microscope (Nikon, Tokyo, Japan) equipped with a Prime BSI Scientific CMOS camera (Photometrics^®^ Prime BSI™, Scottsdale, AZ, USA). Z-stacks were acquired with a 0.3 µm step size, followed by deconvolution using the Richardson–Lucy algorithm (20 iterations) in Nikon NIS-Elements Advanced Research software (version 6.20.00). Maximum intensity projections of the fluorescence signals were generated for visualization. Additionally, confocal imaging was performed on a Zeiss LSM 800 (Observer Z1, Carl Zeiss, Oberkochen, Germany). A Z-stack was acquired with 0.21 µm steps, and 3D reconstruction was performed using ZEN 2.6 software (Blue Edition, v2.6.76.00000; Carl Zeiss, Oberkochen, Germany; file v2.6.18299.3).

### 4.3. Isolation and Culture of L. donovani-iRFP Amastigotes from Mouse Bone Marrow

*L. donovani*-iRFP has been previously used by our group in drug screening experiments [27,75] and is a genetically modified strain derived from *L. donovani* LV9 that constitutively expresses the gene encoding the iRFP of *Rhodopseudomonas palustris* [76,77].

Amastigotes were obtained by infecting BALB/c mice with metacyclic promastigotes of *L. donovani*-iRFP. Briefly, six- to eight-week-old female BALB/c mice were inoculated intraperitoneally with 1.5 × 10^9^ *L. donovani*-iRFP metacyclic promastigotes. Eight weeks post-infection, mice were euthanized and the femurs and tibiae were aseptically collected. The epiphyses were excised, and bone marrow was flushed with PBS using 27G syringes (Terumo, Tokyo, Japan) through 100 µm strainers into 50 mL conical tubes. The cell suspension was centrifuged at 2500 rpm for 10 min, and the supernatant discarded. The resulting pellet was resuspended in 20 mL of amastigote medium (KCl (15 mM), KH_2_PO_4_ (136 mM), K_2_HPO_4_·3H_2_O (10 mM), MgSO_4_·7H_2_O (0.5 mM), NaHCO_3_ (24 mM), glucose (22 mM), L-glutamine (1 mM), vitamins (1× RPMI 1640), folic acid (10 μM), adenosine (100 μM), amino acids (1× RPMI 1640), hemin (5 μg/mL), penicillin (50 U/mL), streptomycin (50 μg/mL), and MES (25 mM) in Milli-Q water, adjusted to pH 5.66 and supplemented with 10% fetal bovine serum (FBS)). Bone marrow cells were incubated at 37 °C with 5% CO_2_ to release the axenic amastigotes, which were subsequently maintained under the same conditions.

Near-infrared fluorescence (700 nm) emitted by viable *L. donovani*-iRFP was measured on an Odyssey infrared imaging system (Li-Cor, Lincoln, NE, USA).

### 4.4. Co-Culture of Hepatic Organoids with Axenic Amastigotes

Co-culture was carried out in 24-well plates. Seven days after organoid culture, the medium was removed, and the Matrigel^®^ matrix (Corning^®^, Discovery Labware Inc., Bedford, MA, USA) domes were gently dissociated with 800 µL of pre-warmed advanced DMEM/F-12. The resulting cell suspension was collected and centrifuged in a 15 mL tube at 290× *g* for 5 min. The cell pellet was resuspended in 35 µL of Matrigel^®^ matrix (Corning^®^, Discovery Labware Inc., Bedford, MA, USA) containing 2 × 10^6^ axenic *L. donovani*-iRFP amastigotes (per well), which were previously washed twice with PBS at 3.500 rpm for 7 min to remove residual amastigote medium. On the other hand, the cell pellet was also resuspended in Matrigel^®^ matrix (Corning^®^, Discovery Labware Inc., Bedford, MA, USA) without amastigotes as control. The co-culture was incubated for 24 h at 37 °C with 5% CO_2_. The pellet was used for RNA isolation (RNAseq analysis), whereas the supernatant was collected for NO and cytokine analysis (see below).

### 4.5. RNAseq Analysis

Transcriptome analysis was carried out by RNAseq. Samples from mouse liver and hepatic organoids were taken as described below.

Four pieces of hepatic tissue (~1 cm) from BALB/c mice were preserved in RNAlater (Fisher Scientific, Waltham, MA, USA) and stored at −80 °C. Organoids and organoids co-cultured with *L. donovani*-iRFP axenic amastigotes were collected from four different wells for each condition. Then, samples were centrifuged at 290× *g* for 5 min at 4 °C resuspended in RNAlater^®^ (Thermo Fisher Scientific, Waltham, MA, USA) and stored at −80 °C as four independent samples.

Frozen samples were shipped to Seqplexing Genetest (Valencia, Spain), where total RNA was extracted from both tissue and organoid samples using a commercial RNA extraction kit according to the manufacturer’s instructions. RNA quality and concentration were assessed with a Quantus Fluorometer (Promega, Madison, WI, USA). Libraries were then prepared and sequenced on an Illumina NovaSeq X platform (2 × 150 bp, paired-end).

Raw sequencing reads were processed bioinformatically. The RNA sequencing quality control confirmed appropriate fragment size, and sequencing quality was assessed with FastQC, providing metrics such as read counts, GC content, and Q20/Q30 scores, confirming the adequacy of these parameters. Reads were aligned to the GRCm39 reference genome using STAR, accounting for transcript splicing, and duplicate reads were removed with UMI-tools. Post-mapping quality control was performed using HTSeq to quantify reads mapped to each gene, and most samples had over 90% of reads successfully aligned. Gene expression was quantified using HTSeq-count, normalized with DESeq2, and differential expression was assessed with False Discovery Rate correction according to the Benjamini–Hochberg method. Data visualization was performed in R (version 4.4.2) using packages such as ggplot2 (version 3.5.1) and pheatmap (version 1.0.12) to generate heatmaps, Volcano plots, Principal Component Analysis (PCA) plots, and dispersion curves.

Functional enrichment analyses, including KEGG pathway (biological, cellular and molecular function) were conducted using DAVID server [25,26] of the NIH (DAVID Functional Annotation Bioinformatics Microarray Analysis; accessed on 17 September 2025). Differentially expressed genes were classified as upregulated or downregulated based on a Log2FC < −1 or >1, respectively, and a q-value < 0.1. Gene identifiers were standardized to ENTREZ Gene IDs, achieving a conversion success rate exceeding 99%.

### 4.6. Cytotoxicity Assays

Cytotoxicity assays were carried out in 384-well plates. HepG2 cells were cultured in DMEM/F-12 (Gibco, Life Technologies Limited, Paisley, UK) supplemented with 10% FBS (Gibco, Thermo Fisher Scientific, Waltham, MA, USA) and 1% penicillin/streptomycin (Hyclone, Thermo Fisher Scientific, Waltham, MA, USA), while RAW 264.7 cells were maintained in RPMI medium supplemented with 10% FBS. Cells (2500 cells in 40 µL of the specific medium for each cell line) were allowed to adhere to the bottom of the wells for 24 h at 37 °C in a 5% CO_2_. Then, 10 µL of different concentrations (from 150 μM to 0.05 μM using 2/3 serial dilutions) of 5-nitro-2-furonitrile were added to each well and the culture was incubated for 72 h under the same incubation conditions. As positive control 0.1% H_2_O_2_ was used, whereas 0.1% DMSO was used as the negative control.

Mouse intestinal organoids were developed and grown as previously described [28,42]. Fully developed mouse hepatic and intestinal organoids (day 4 of growth) in 40 μL of medium were treated with 10 µL of different concentrations (from 300 μM to 0.09 μM using 2/3 serial dilutions) of 5-nitro-2-furonitrile and the culture was incubated for 72 h at 37 °C in a 5% CO_2_. As positive control 0.03% H_2_O_2_ was used, whereas 0.2% DMSO was used as the negative control.

Cell viability was assessed by adding alamarBlue™ Cell Viability Reagent (10% *v*/*v*; Invitrogen™, Thermo Fisher Scientific™, Waltham, MA, USA) to each well. After either 4 h (for HepG2 and RAW 264.7 cells) or 24 h (for organoids) of incubation at 37 °C with 5% CO_2_, fluorescence was quantified with a Varioskan™ LUX microplate reader (Thermo Scientific, Waltham, MA, USA; Instrument software v1.00.37; SkanIt software v5.0.0.42). The CC_50_ values were determined by nonlinear regression analysis using SigmaPlot^®^ 10.0 (Grafiti LLC, Austin, TX, USA, built 10.0.0.54). Data from the negative control were set as 100% viability, whereas data from the positive control was set as 0% viability.

### 4.7. Analysis of NO Production

NO production was determined by measuring nitrite accumulation in cell culture supernatants using the Griess reagent. The reagent was prepared by mixing equal 1/1 volumes of Solution A (0.1% naphthylethylenediamine dihydrochloride (Sigma-Aldrich, St. Louis, MO, USA) in distilled water) and Solution B (1% sulfanilamide (Panreac, Barcelona, Spain) in 5% orthophosphoric acid (Acros Organics, Geel, Belgium). The standard curve was prepared from a 50 mM NaNO_2_ stock solution by performing serial 1:2 dilutions in culture medium. In 96-well plates, 50 µL of standards or samples were mixed with 50 µL of freshly prepared Griess reagent. Plates were incubated for 10 min at room temperature in the dark, and absorbance was measured at 550 nm in a Varioskan™ LUX microplate reader (Thermo Scientific, Waltham, MA, USA; Instrument software v1.00.37; SkanIt software v5.0.0.42).

### 4.8. Cytokine Analysis

The Olink^®^ Target 48 Mouse Cytokine Panel (Olink Proteomics, Uppsala, Sweden) was applied to analyze the supernatants of organoids and organoids co-cultured with *L. donovani*-iRFP amastigotes. The analysis was carried out by Cobiomic Bioscience (Córdoba, Spain). Three technical replicates were analyzed for each condition.

### 4.9. Statistical Analysis

To quantify organoid growth over time, images were captured from a randomly selected well of a culture plate over 5 days. The largest diameter of 21 randomly selected organoids at each time point was measured by confocal microscopy (see above) and data were analyzed in RStudio v2024.12.1+563 (Auckland, CA, USA). Normality was assessed using the Kolmogorov–Smirnov and Shapiro–Wilk tests in GraphPad Prism 8 (GraphPad Software, San Diego, CA, USA). Group differences were analyzed using an unpaired, two-tailed Student’s *t*-test.

The CC_50_ values were determined by nonlinear regression analysis using SigmaPlot^®^ 10.0 (Grafiti LLC, Austin, TX, USA, built 10.0.0.54).

HTS robustness and reliability were assessed by calculating key statistical metrics including the Z′-factor, signal-to-background (S/B) ratio, signal-to-noise (S/N) ratio, SW, and AVR, following the guidelines of [78]. The Z′-factor [79] was determined in a 2 × 2 format (four independent experiments in 384-well plates), using the values provided by 0.03% H_2_O_2_ as the minimum signal and the values provided by 0.2% DMSO as the maximum signal.

## 5. Conclusions

Data presented in this work demonstrate that mouse hepatic organoids constitute a versatile and physiologically relevant model for studying toxicity, tissue response, and complex infectious processes. The dual response—regenerative activation and immune repression—suggests that the organoids faithfully reproduce the evasion and remodeling mechanisms that *L. donovani* establishes in vivo during VL.

Although mouse hepatic organoids do not fully recapitulate all aspects of mature liver physiology, including certain metabolic and signaling pathways, they retain key functional and transcriptional programs that allow them to serve as a practical and robust model. Therefore, despite these intrinsic limitations, organoids provide a valuable platform for mechanistic studies, cell–cell interaction analyses, and antiparasitic drug screening in a 3D context that overcomes many constraints of traditional 2D cultures.

Nevertheless, several limitations should be considered when interpreting these results, such as the absence of other key cell types of the hepatic microenvironment (Kupffer cells, stellate cells, and sinusoidal endothelial cells) as well as the lack of endocrine signals, vascularization, or the metabolic gradients characteristic of the physiological liver. Notwithstanding these limitations, this system opens the door for future application in mechanistic studies, cell–cell interaction analysis, and antiparasitic drug screening in a controlled and physiologically relevant 3D environment.

## Figures and Tables

**Table 1:** Top six most upregulated genes in mouse liver versus mouse hepatic organoids, ranked by *p*-value.

Gene Product	Description ^1^	Log2FC ^2^	*p*-Value
Cdo1	Cysteine dioxygenase 1, cytosolic. Predicted to be involved in several processes, including L-cysteine catabolic process; lactation; and response to glucagon. Predicted to act upstream of or within L-cysteine catabolic process to taurine.	6.5	5.2 × 10^−274^
Elovl5	ELOVL fatty acid elongase 5. Involved in fatty acid derivative biosynthetic process and fatty acid metabolic process.	3.9	2.0 × 10^−247^
Cat	Catalase. It acts in several processes, including hydrogen peroxide catabolic process; positive regulation of phosphatidylinositol 3-kinase/protein kinase B signal transduction; and regulation of DNA-binding transcription factor activity.	5.1	1.4 × 10^−236^
Ndrg2	N-myc downstream regulated gene 2. It acts upstream of or within negative regulation of ERK1 and ERK2 cascade; negative regulation of vascular associated smooth muscle cell proliferation; and regulation of cytokine production.	5.8	3.0 × 10^−233^
Hmgcs2	3-hydroxy-3-methylglutaryl-Coenzyme A synthase 2. Predicted to be involved in acetyl-CoA metabolic process; farnesyl diphosphate biosynthetic process, mevalonate pathway; and ketone body biosynthetic process.	5.6	2.6 × 10^−230^
Phyh	Phytanoyl-CoA hydroxylase. It acts upstream of or within fatty acid alpha-oxidation.	4.6	1.6 × 10^−193^

^1^ Information summarized from the National Library of Medicine (https://www.ncbi.nlm.nih.gov/datasets/gene/; accessed on 17 September 2025). ^2^ Log2FC = Log2 of the calculated fold change.

**Table 2:** Top six most downregulated genes in mouse liver versus mouse hepatic organoids, ranked by *p*-value.

Gene Product	Description ^1^	Log2FC ^2^	*p*-Value
Cdh1	Cadherin 1. Calcium-dependent cell adhesion molecule that participates in the establishment and maintenance of epithelial cell morphology during embryogenesis and adulthood.	−3.6	0.0
D17H6S56E-5	DNA segment, Chr 17, human D6S56E 5.	−6.3	0.0
Cd44	CD44 antigen. It enables cargo receptor activity; hyaluronic acid binding activity; and type II transforming growth factor beta receptor binding activity. It contributes to cytokine binding activity and cytokine receptor activity. Involved in hyaluronan catabolic process; negative regulation of T cell activation; and regulation of intracellular signal transduction. It acts in Wnt signaling pathway; morphogenesis of a branching epithelium; and wound healing involved in inflammatory response.	−6.6	1.8 × 10^−257^
LOC118568312	Long non-coding RNA (lncRNA).	−6.9	8.9 × 10^−236^
S100a6	S100 calcium binding protein A6 (calcyclin). It enables calcium ion binding activity and zinc ion binding activity. Predicted to be involved in monoatomic ion transmembrane transport.	−7.3	7.5 × 10^−223^
Malat1	Metastasis associated lung adenocarcinoma transcript 1 (lncRNA). This lncRNA is cleaved into a mature, functional transcript that functions in the regulation of gene expression and proliferation and may be upregulated in tumors.	−5.4	2.9 × 10^−214^

^1^ Information summarized from the National Library of Medicine (https://www.ncbi.nlm.nih.gov/datasets/gene/ accessed on 17 September 2025). ^2^ Log2FC = Log2 of the calculated fold change.

**Table 3:** Metrics and average statistical values from four independent experiments obtained from Z′-factor tests. Optimal reference values: Z′ ≥ 0.5, S/B > 5, S/N > 10, SW > 2, AVR < 0.5.

Metrics ^1^	Average Value ^2^
Z′	0.7
S/B	37.6
S/N	208.0
SW	8.0
AVR	0.3

^1^ AVR: Assay Variability Ratio; S/B: Signal-to-Background ratio; S/N: Signal-to-Noise ratio; SW: Signal Window. ^2^ Mean values from four independent experiments (2 × 2 format).

**Table 4:** Top six most overexpressed genes in hepatic organoids after co-culture with *L. donovani*-iRFP, ranked by *p*-value.

Gene Product	Description ^1^	Log2FC ^2^	*p*-Value
Ankrd1	Ankyrin repeat domain 1. Involved in cellular response to lipopolysaccharide and cellular response to xenobiotic stimulus.	2.5	5.3 × 10^−67^
Klf6	Kruppel-like transcription factor 6. Predicted to enable DNA-binding transcription activator activity, RNA polymerase II-specific and RNA polymerase II cis-regulatory region sequence-specific DNA binding activity. Involved in positive regulation of connective tissue replacement.	1.5	7.1 × 10^−31^
Top2a	Topoisomerase (DNA) II alpha. It enables DNA topoisomerase type II (double strand cut, ATP-hydrolyzing) activity. Involved in chromosome organization, embryonic cleavage, and positive regulation of transcription by RNA polymerase II.	1.6	2.1 × 10^−29^
Pclaf	PCNA clamp associated factor. Predicted to be involved in several processes, including DNA metabolic process, centrosome cycle, and response to UV.	1.9	2.7 × 10^−26^
Birc5	Survivin. Member of the inhibitor of apoptosis (IAP) gene family, which encode negative regulatory proteins that prevent apoptotic cell death.	1.7	2.3 × 10^−25^
Emp1	Epithelial membrane protein 1. Predicted to be involved in apoptotic process and bleb assembly.	2.7	1.7 × 10^−22^

^1^ Information summarized from the National Library of Medicine (https://www.ncbi.nlm.nih.gov/datasets/gene/ accessed on 17 September 2025). ^2^ Log2FC = Log2 of the calculated fold change.

**Table 5:** Top six most downregulated genes in hepatic organoids after co-culture with *L. donovani*-iRFP, ranked by *p*-value.

Gene Product	Description ^1^	Log2FC ^2^	*p*-Value
Ifitm3	Interferon-induced transmembrane protein 3. Involved in host-mediated suppression of symbiont invasion and response to virus. It acts upstream of or within defense response to other organisms, negative regulation of cell population proliferation, and receptor-mediated endocytosis.	−2.0	4.4 × 10^−35^
Ly6a	Lymphocyte antigen 6 family member A also known as stem cell antigen 1 (Sca-1). Predicted to enable acetylcholine receptor binding activity and acetylcholine receptor inhibitor activity. Acts upstream of or within response to bacteria.	−2.6	1.3 × 10^−33^
Trf	Transferrin. Predicted to enable iron chaperone activity, iron ion binding activity, and transferrin receptor binding activity. Involved in ERK1 and ERK2 cascade; positive regulation of bone resorption; and positive regulation of metabolic process. Acts upstream of or within iron ion transport and response to bacterium.	−2.6	1.4 × 10^−29^
Ifi44	Interferon-induced protein 44. It acts upstream of or within cellular response to virus and bacteria.	−3.7	3.6 × 10^−26^
Isg15	Interferon-stimulated gene 15 ubiquitin-like modifier. Involved in ISG15-protein conjugation, defense response to other organisms and positive regulation of erythrocyte differentiation. It acts upstream of or within modification-dependent protein catabolic process and in response to bacteria.	−2.8	3.4 × 10^−21^
Gsta3	Glutathione S-transferase alpha 3. Involved in ureteric bud development. It acts upstream of or within aflatoxin catabolic process.	−1.9	6.5 × 10^−21^

^1^ Information summarized from the National Library of Medicine (https://www.ncbi.nlm.nih.gov/datasets/gene/ accessed on 17 September 2025). ^2^ Log2FC = Log2 of the calculated fold change.

## Data Availability

Data supporting reported results can be found in the Zenodo repository at https://doi.org/10.5281/zenodo.17791912.

## Supplementary Materials

The following supporting information can be downloaded at: https://www.mdpi.com/article/10.3390/ijms262412180/s1.

## Abbreviations

The following abbreviations are used in this manuscript:

2D	Two-dimensional
3D	Three-dimensional
FBS	Fetal bovine serum
H&E	Hematoxylin and eosin
HTS	High-throughput system
iRFP	Infrared fluorescent protein
KEGG	Kyoto Encyclopedia of Genes and Genomes
PAS	Periodic Acid–Schiff
PBS	Phosphate-buffered saline
RT	Room temperature
VL	Visceral leishmaniasis
ZO	Zonula occludens

## References

[1] Urzì O., Gasparro R., Costanzo E., De Luca A., Giavaresi G., Fontana S., Alessandro R. (2023). Three-Dimensional cell cultures: The bridge between in vitro and in vivo models. Int. J. Mol. Sci..

[2] Zhao Z., Chen X., Dowbaj A.M., Sljukic A., Bratlie K., Lin L., Fong E.L.S., Balachander G.M., Chen Z., Soragni A. (2022). Organoids. Nat. Rev. Methods Primers.

[3] Kim M.B., Hwangbo S., Jang S., Jo Y.K. (2022). Bioengineered co-culture of organoids to recapitulate host–microbe interactions. Mater. Today Bio.

[4] Zhang Y., Liu T., He W. (2024). The application of organoids in cancers associated with pathogenic infections. Clin. Exp. Med..

[5] Yao Q., Cheng S., Pan Q., Yu J., Cao G., Li L., Cao H. (2024). Organoids: Development and applications in disease models, drug discovery, precision medicine, and regenerative medicine. MedComm.

[6] Naderi-Meshkin H., Cornelius V.A., Eleftheriadou M., Potel K.N., Setyaningsih W.A.W., Margariti A. (2023). Vascular organoids: Unveiling advantages, applications, challenges, and disease modelling strategies. Stem Cell Res. Ther..

[7] Yip S., Wang N., Sugimura R. (2023). Give them vasculature and immune cells: How to fill the gap of organoids. Cells Tissues Organs.

[8] Li K., He Y., Jin X., Jin K., Qian J. (2025). Reproducible extracellular matrices for tumor organoid culture: Challenges and opportunities. J. Transl. Med..

[9] Fatehullah A., Tan S.H., Barker N. (2016). Organoids as an in vitro model of human development and disease. Nat. Cell Biol..

[10] Kar S.K., Wells J.M., Ellen E.D., Te Pas M.F.W., Madsen O., Groenen M.A.M., Woelders H. (2021). Organoids: A promising new in vitro platform in livestock and veterinary research. Vet. Res..

[11] Kim H.M., Kim Y., Kim Y., Kim Y.J., Ko K.S. (2023). Organoid establishment of long-term culture using primary mouse hepatocytes and evaluation of liver function. Prev. Nutr. Food Sci..

[12] Zhou Z., Zheng X., Xie M., Lin Z., Du F., Shi X., Li R. (2024). Mice hepatic organoids for modeling nonalcoholic fatty liver disease and drug response. Stem Cells Dev..

[13] Brooks A., Liang X., Zhang Y., Zhao C.X., Roberts M.S., Wang H., Zhang L., Crawford D.H.G. (2021). Liver organoid as a 3D in vitro model for drug validation and toxicity assessment. Pharmacol. Res..

[14] Burza S., Croft S.L., Boelaert M. (2018). Leishmaniasis. Lancet.

[15] Pareyn M., Alves F., Burza S., Chakravarty J., Alvar J., Diro E., Kaye P.M., van Griensven J. (2025). Leishmaniasis. Nat. Rev. Dis. Primers.

[16] WHO Leishmaniasis. https://www.who.int/news-room/fact-sheets/detail/leishmaniasis.

[17] Rodrigues V., Cordeiro-da-Silva A., Laforge M., Silvestre R., Estaquier J. (2016). Regulation of immunity during visceral *Leishmania* infection. Parasit. Vectors.

[18] Croft S.L., Sundar S., Fairlamb A.H. (2006). Drug resistance in leishmaniasis. Clin. Microbiol. Rev..

[19] Frézard F., Demicheli C., Ribeiro R.R. (2009). Pentavalent antimonials: New perspectives for old drugs. Molecules.

[20] van Griensven J., Diro E. (2019). Visceral leishmaniasis: Recent advances in diagnostics and treatment regimens. Infect. Dis. Clin. N. Am..

[21] Reguera R.M., Pérez-Pertejo Y., Gutiérrez-Corbo C., Domínguez-Asenjo B., Ordóñez C., García-Estrada C., Martínez-Valladares M., Balaña-Fouce R. (2019). Current and promising novel drug candidates against visceral leishmaniasis. Pure Appl. Chem..

[22] Pinheiro A.C., de Souza M.V.N. (2022). Current leishmaniasis drug discovery. RSC Med. Chem..

[23] Ramli M.N.B., Lim Y.S., Koe C.T., Demircioglu D., Tng W., Gonzales K.A.U., Tan C.P., Szczerbinska I., Liang H., Soe E.L. (2020). Human pluripotent stem cell-derived organoids as models of liver disease. Gastroenterology.

[24] Fiorotto R., Mariotti V., Taleb S.A., Zehra S.A., Nguyen M., Amenduni M., Strazzabosco M. (2023). Cell-matrix interactions control biliary organoid polarity, architecture, and differentiation. Hepatol. Commun..

[25] Huang D.W., Sherman B.T., Lempicki R.A. (2009). Systematic and integrative analysis of large gene lists using DAVID bioinformatics resources. Nat. Protoc..

[26] Sherman B.T., Hao M., Qiu J., Jiao X., Baseler M.W., Lane H.C., Imamichi T., Chang W. (2022). DAVID: A web server for functional enrichment analysis and functional annotation of gene lists (2021 Update). Nucleic Acids Res..

[27] Andrés-Rodríguez J., González-Montero M.C., García-Fernández N., Galano-Frutos J.J., Rosa M.C., Ferreira P., Pérez-Pertejo M.Y., Reguera R.M., Balaña-Fouce R., García-Estrada C. (2025). Mechanistic, in-silico and in vitro studies with nitrofurans reveal potent leishmanicidal activity and inhibition of trypanothione reductase. Int. J. Parasitol. Drugs Drug Resist..

[28] Galli G., Ruiz-Somacarrera M., González Del Palacio L., Melcón-Fernández E., González-Pérez R., García-Estrada C., Martinez-Valladares M., Balaña-Fouce R. (2025). High-throughput screening of five compound libraries for anthelmintic activity and toxicity leads to the discovery of two flavonoid compounds. Int. J. Mol. Sci..

[29] Gopallawa I., Gupta C., Jawa R., Cyril A., Jawa V., Chirmule N., Gujar V. (2024). Applications of organoids in advancing drug discovery and development. J. Pharm. Sci..

[30] Morizane R., Lamers M.M. (2025). Organoids in disease modeling and regenerative medicine. Cell. Mol. Life Sci..

[31] Huch M., Dorrell C., Boj S.F., van Es J.H., Li V.S., van de Wetering M., Sato T., Hamer K., Sasaki N., Finegold M.J. (2013). In vitro expansion of single lgr5^+^ liver stem cells induced by wnt-driven regeneration. Nature.

[32] Hu H., Gehart H., Artegiani B., López-Iglesias C., Dekkers F., Basak O., van Es J., Chuva de Sousa Lopes S.M., Begthel H., Korving J. (2018). Long-term expansion of functional mouse and human hepatocytes as 3D organoids. Cell.

[33] Tomofuji K., Kondo J., Onuma K., Coppo R., Horie H., Oyama K., Miyoshi E., Fukumitsu K., Ishii T., Hatano E. (2023). Hepatocyte differentiation from mouse liver ductal organoids by transducing 4 liver-specific transcription factors. Hepatol. Commun..

[34] Cohn J.A., Strong T.V., Picciotto M.R., Nairn A.C., Collins F.S., Fitz J.G. (1993). Localization of the cystic fibrosis transmembrane conductance regulator in human bile-duct epithelial cells. Gastroenterology.

[35] Messner S., Fredriksson L., Lauschke V.M., Roessger K., Escher C., Bober M., Kelm J.M., Ingelman-Sundberg M., Moritz W. (2018). Transcriptomic, proteomic, and functional long-term characterization of multicellular three-dimensional human liver microtissues. Appl. Vitr. Toxicol..

[36] Ariño S., Ferrer-Lorente R., Serrano G., Zanatto L., Martínez-García de la Torre R.A., Gratacós-Ginès J., Rubio A.B., Pérez M., Mateos-Sánchez C., Aguilar-Bravo B. (2025). Patient-derived liver organoids recapitulate liver epithelial heterogeneity and enable precision modeling of alcohol-related liver disease. J. Hepatol..

[37] Sun X.C., Kong D.F., Zhao J., Faber K.N., Xia Q., He K. (2023). Liver organoids: Established tools for disease modeling and drug development. Hepatol. Commun..

[38] Liu W., Ye H. (2014). Co-expression network analysis identifies transcriptional modules in the mouse liver. Mol. Genet. Genomics.

[39] Grant D.M. (1991). Detoxification pathways in the liver. J. Inherit. Metab. Dis..

[40] Xiong C., Baker D., Pietrantonio P. (2022). A “Dual-addition” calcium fluorescence assay for the high-throughput screening of recombinant g protein-coupled receptors. J. Vis. Exp..

[41] Carr A.J., Hajicek N., Tsai A.P., Acharya P.P., Hardy P.B., Meyer E., Wyss-Coray T., Pearce K.H., Sondek J., Zhang Q. (2025). A high-throughput assay platform to discover small molecule activators of the phospholipase PLC-γ2 to treat alzheimer’s disease. J. Biol. Chem..

[42] Galli G., Melcón-Fernández E., de Garnica García M.G., Martínez-Fernández B., Dehnavi M., Andrés S., Pérez-Pertejo Y., Reguera R.M., García-Estrada C., Martínez-Valladares M. (2025). Development of sheep duodenum intestinal organoids and implementation of high-throughput screening platform for veterinary applications. Int. J. Mol. Sci..

[43] Suarez-Martinez E., Suazo-Sanchez I., Celis-Romero M., Carnero A. (2022). 3D and organoid culture in research: Physiology, hereditary genetic diseases and cancer. Cell Biosci..

[44] Caipa Garcia A.L., Arlt V.M., Phillips D.H. (2022). Organoids for toxicology and genetic toxicology: Applications with drugs and prospects for environmental carcinogenesis. Mutagenesis.

[45] Park E., Kim H.K., Jee J., Hahn S., Jeong S., Yoo J. (2019). Development of organoid-based drug metabolism model. Toxicol. Appl. Pharmacol..

[46] Caipa Garcia A.L., Kucab J.E., Al-Serori H., Beck R.S.S., Fischer F., Hufnagel M., Hartwig A., Floeder A., Balbo S., Francies H. (2022). Metabolic activation of benzo[a]pyrene by human tissue organoid cultures. Int. J. Mol. Sci..

[47] Rodrigues A.V., Alexandre-Pires G., Valério-Bolas A., Santos-Mateus D., Rafael-Fernandes M., Pereira M.A., Ligeiro D., Nunes T., Alves-Azevedo R., Santos M. (2020). 3D-hepatocyte culture applied to parasitology: Immune activation of canine hepatic spheroids exposed to *Leishmania infantum*. Biomedicines.

[48] Heo I., Dutta D., Schaefer D.A., Iakobachvili N., Artegiani B., Sachs N., Boonekamp K.E., Bowden G., Hendrickx A.P.A., Willems R.J.L. (2018). Modelling *Cryptosporidium* infection in human small intestinal and lung organoids. Nat. Microbiol..

[49] Seo H.H., Han H.W., Lee S.E., Hong S.H., Cho S.H., Kim S.C., Koo S.K., Kim J.H. (2020). Modelling *Toxoplasma gondii* infection in human cerebral organoids. Emerg. Microbes Infect..

[50] Shpichka A., Bikmulina P., Peshkova M., Heydari Z., Kosheleva N., Vosough M., Timashev P. (2022). Organoids in modelling infectious diseases. Drug Discov. Today.

[51] Nitaramorn N., Kobpornchai P., Tongkrajang N., Chaisri U., Imwong M., Kulkeaw K. (2024). Human liver organoids are susceptible to *Plasmodium vivax* Infection. Malar. J..

[52] Bogdan C., Islam N.A., Barinberg D., Soulat D., Schleicher U., Rai B. (2024). The Immunomicrotope of *Leishmania* control and persistence. Trends Parasitol..

[53] Sadler K.C., Krahn K.N., Gaur N.A., Ukomadu C. (2007). Liver growth in the embryo and during liver regeneration in zebrafish requires the cell cycle regulator, Uhrf1. Proc. Natl. Acad. Sci. USA.

[54] Baba H.A., Wohlschlaeger J., Schmitz K.J., Nadalin S., Lang H., Benesch A., Gu Y., Biglarnia A.R., Sotiropoulos G.C., Takeda A. (2009). Survivin is upregulated during liver regeneration in rats and humans and is associated with hepatocyte proliferation. Liver Int..

[55] Sydor S., Manka P., Best J., Jafoui S., Sowa J.P., Zoubek M.E., Hernandez-Gea V., Cubero F.J., Kälsch J., Vetter D. (2017). Krüppel-Like Factor 6 is a transcriptional activator of autophagy in acute liver injury. Sci. Rep..

[56] Santamaría E., Rodríguez-Ortigosa C.M., Uriarte I., Latasa M.U., Urtasun R., Alvarez-Sola G., Bárcena-Varela M., Colyn L., Arcelus S., Jiménez M. (2019). The epidermal growth factor receptor ligand amphiregulin protects from cholestatic liver injury and regulates bile acids synthesis. Hepatology.

[57] Rostan O., Gangneux J.P., Piquet-Pellorce C., Manuel C., McKenzie A.N., Guiguen C., Samson M., Robert-Gangneux F. (2013). The IL-33/ST2 axis is associated with human visceral leishmaniasis and suppresses Th1 responses in the livers of BALB/c mice infected with *Leishmania donovani*. mBio.

[58] Roy S., Roy S., Halder S., Jana K., Ukil A. (2024). *Leishmania* Exploits Host cAMP/EPAC/Calcineurin signaling to induce an IL-33-mediated anti-inflammatory environment for the establishment of infection. J. Biol. Chem..

[59] Cepero-Donates Y., Rakotoarivelo V., Mayhue M., Ma A., Chen Y.G., Ramanathan S. (2016). Homeostasis of IL-15 dependent lymphocyte subsets in the liver. Cytokine.

[60] Jiao J., Ooka K., Fey H., Fiel M.I., Rahmman A.H., Kojima K., Hoshida Y., Chen X., de Paula T., Vetter D. (2016). Interleukin-15 receptor α on hepatic stellate cells regulates hepatic fibrogenesis in mice. J. Hepatol..

[61] Xin L., Vargas-Inchaustegui D.A., Raimer S.S., Kelly B.C., Hu J., Zhu L., Sun J., Soong L. (2010). Type I IFN Receptor regulates neutrophil functions and innate immunity to *Leishmania* parasites. J. Immunol..

[62] Dias B.T., Goundry A., Vivarini A.C., Costa T.F.R., Mottram J.C., Lopes U.G., Lima A.P.C.A. (2022). Toll-Like Receptor- and Protein Kinase R-induced Type I interferon sustains infection of *Leishmania donovani* in macrophages. Front. Immunol..

[63] Ruhland A., Leal N., Kima P.E. (2007). *Leishmania* promastigotes activate PI3K/Akt signalling to confer host cell resistance to apoptosis. Cell. Microbiol..

[64] Filho A.A.P., Nascimento A.A.S., Saab N.A.A., Fugiwara R.T., D’Ávila Pessoa G.C., Koerich L.B., Pereira M.H., Araújo R.N., Sant’Anna M.R.V., Gontijo N.F. (2021). Evasion of the complement system by *Leishmania* through the uptake of factor H, a complement regulatory protein. Acta Trop..

[65] Pereira-Filho A.A., Queiroz D.C., Saab N.A.A., D’Ávila Pessoa G.C., Koerich L.B., Pereira M.H., Sant’Anna M.R.V., Araújo R.N., Bartholomeu D.C., Gontijo N.F. (2023). Evasion of the complement system by *Leishmania* through the uptake of C4bBP, a complement regulatory protein, and probably by the action of GP63 on C4b molecules deposited on parasite surface. Acta Trop..

[66] Olivier M., Gregory D.J., Forget G. (2005). Subversion mechanisms by which *Leishmania* parasites can escape the host immune response: A signaling point of view. Clin. Microbiol. Rev..

[67] Lerzynski G., Suschek C.V., Kolb-Bachofen V. (2006). In hepatocytes the regulation of NOS-2 activity at physiological L-arginine levels suggests a close link to the urea cycle. Nitric Oxide.

[68] Pessenda G., da Silva J.S. (2020). Arginase and its mechanisms in *Leishmania* persistence. Parasite Immunol..

[69] Saha A., Roy S., Ukil A. (2022). Cytokines and signaling networks regulating disease outcomes in leishmaniasis. Infect. Immun..

[70] Faleiro R.J., Kumar R., Hafner L.M., Engwerda C.R. (2014). Immune regulation during chronic visceral leishmaniasis. PLoS Negl. Trop. Dis..

[71] Rodrigues A., Alexandre Pires G., Valério Bolas A., Nunes T., Pereira da Fonseca I., Santos Gomes G. (2024). Kupffer cells and hepatocytes: A key relation in the context of canine leishmaniasis. Microorganisms.

[72] Document #10000003511. Version 03. HepatiCult^TM^ Organoid Growth Medium (Mouse): Cell Culture Medium for Establishment and Maintenance of Mouse Hepatic Progenitor Organoids. https://cdn.stemcell.com/media/files/pis/10000003511-PIS_03.pdf.

[73] Document #27087. Version 1.0.0. December 2017. Technical Bulletin: Mouse Hepatic Progenitor Organoid Culture: Supplementary Protocol. https://cdn.stemcell.com/media/files/techbulletin/TB27087-Mouse_Hepatic_Progenitor_Organoid_Culture.pdf.

[74] Fujii E., Yamazaki M., Kawai S., Ohtani Y., Watanabe T., Kato A., Suzuki M. (2018). A simple method for histopathological evaluation of organoids. J. Toxicol. Pathol..

[75] Melcón-Fernández E., Galli G., García-Estrada C., Balaña-Fouce R., Reguera R.M., Pérez-Pertejo Y. (2023). Miltefosine and nifuratel combination: A promising therapy for the treatment of *Leishmania donovani* visceral leishmaniasis. Int. J. Mol. Sci..

[76] Calvo-Álvarez E., Stamatakis K., Punzón C., Álvarez-Velilla R., Tejería A., Escudero-Martínez J.M., Pérez-Pertejo Y., Fresno M., Balaña-Fouce R., Reguera R.M. (2015). Infrared fluorescent imaging as a potent tool for in vitro, ex vivo and in vivo models of visceral leishmaniasis. PLoS Negl. Trop. Dis..

[77] Carballeira N.M., Montano N., Amador L.A., Rodríguez A.D., Golovko M.Y., Golovko S.A., Reguera R.M., Álvarez-Velilla R., Balaña-Fouce R. (2016). Novel very long-chain α-methoxylated Δ5,9 fatty acids from the sponge *Asteropus niger* are effective inhibitors of Topoisomerases IB. Lipids.

[78] Iversen P.W., Eastwood B.J., Sittampalam G.S., Cox K.L. (2006). A Comparison of assay performance measures in screening assays: Signal window, Z′ factor, and assay variability ratio. J. Biomol. Screen..

[79] Zhang J.-H., Chung T.D.Y., Oldenburg K.R. (1999). A simple statistical parameter for use in evaluation and validation of High Throughput Screening Assays. J. Biomol. Screen..

